# Retraction Note: Association between the composite dietary antioxidant index and metabolic dysfunction-associated steatotic liver disease in adults: a cross-sectional study from NHANES 2017–2020

**DOI:** 10.1038/s41598-025-88794-8

**Published:** 2025-02-11

**Authors:** Zheng Yang, Shupeng Song, Lufeng Li, Zhe Yuan, Yongguo Li

**Affiliations:** 1 Department of Infectious Diseases, The First Affiliated Hospital of Chongqing Medical University, Chongqing, China; 2 Department of Infectious Disease, Jingzhou Hospital Affiliated to Yangtze University, Jingzhou, China

Retraction of:* Scientific Reports* 10.1038/s41598-024-63965-1, published online 14 June 2024

The Authors have retracted this Article.

After publication it was brought to the Authors’ attention that they used two NHANES datasets (2017–2018 and 2017-March2020) that contained partially overlapping data and therefore could not be combined. The Authors performed a reanalysis using the separate NHANES 2017–2018 and 2019-March2020 datasets; however, this new analysis resulted in differences in several significance values, which changed the conclusions of the original Article.

All Authors agree to this retraction and its wording.

